# PREDICTORS OF DEPRESSION AMONG MIDDLE-AGED AND OLDER MEN AND WOMEN IN EUROPE: A MACHINE LEARNING APPROACH

**DOI:** 10.1093/geroni/igac059.716

**Published:** 2022-12-20

**Authors:** Stephen Aichele, Elizabeth Handing, Carolin Strobl, Yuqin Jiao, Leilani Feliciano

**Affiliations:** Colorado State University, Fort Collins, Colorado, United States; Colorado State University, Golden, Colorado, United States; University of Zurich, Zurich, Zurich, Switzerland; Colorado State University at Fort Collins, Fort Collins, Colorado, United States; University of Colorado Colorado Springs, Colorado Springs, Colorado, United States

## Abstract

The high prevalence of depression in a growing aging population represents a critical public health issue. It is unclear how social, health, cognitive, and functional variables rank as risk/protective factors for depression among older adults and whether there are conspicuous differences among men and women. We utilized random forest analysis (RFA), a machine learning method, to compare 56 risk/protective factors for depression in a large representative sample of European older adults (N = 67,603; ages 45-105y; 56.1% women; 18 countries represented) from the Survey of Health, Ageing and Retirement in Europe (SHARE Wave 6). Self-rated social isolation and self-rated poor health were the most salient risk factors, jointly accounting for 22.0% (in men) and 22.3% (in women) of variability in depression. Difficulties in mobility (in both sexes), difficulties in instrumental activities of daily living (in men), and higher self-rated family burden (in women) accounted for an additional but small percentage of variance in depression risk (2.2% in men, 1.5% in women). To our knowledge, this is the first large, multi-country study to use machine learning to compare a broad range of social, health, functional, and cognitive variables as concurrent risk/protective factors for depression in middle-aged and older adults—and with analyses conducted independently for women and men. The results point to the importance of screening for depression risk within this age demographic during routine medical visits (i.e., when assessing general health status) and further indicate that such screening may be improved by inclusion of measures of perceived social isolation.

